# Supplementary material for: Mapping and modeling human colorectal carcinoma interactions with the tumor microenvironment
Source: Nat Commun. 2023 Nov 30;14:7915. doi: 10.1038/s41467-023-43746-6 (PMC10689473; doi:10.1038/s41467-023-43746-6)
Supplement: Supplementary file 1 — Supplementary information [file 41467_2023_43746_MOESM1_ESM.pdf]

Supplemental Figure 1

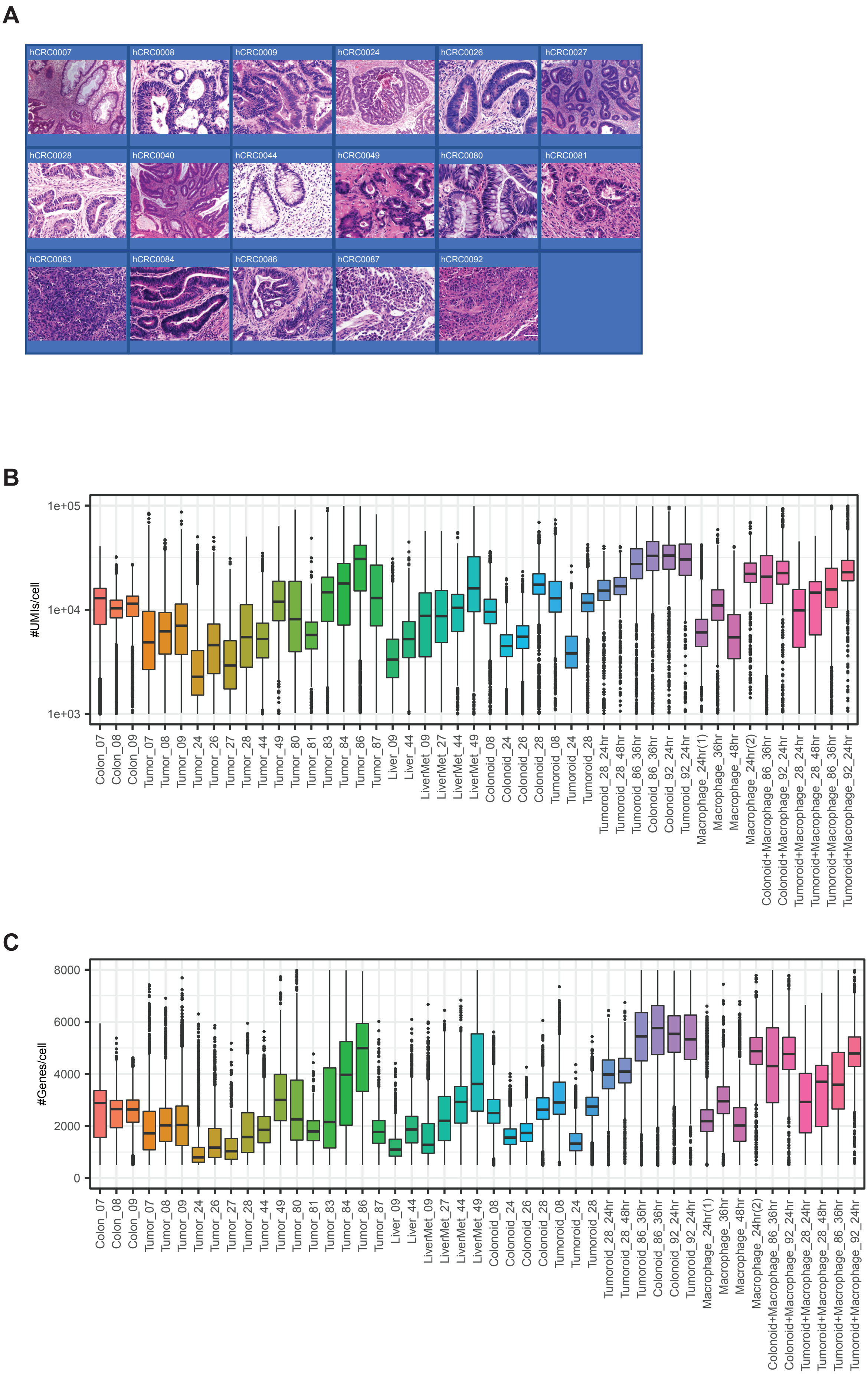

Supplemental Figure 1. Single cell transcriptomic profiling of primary colorectal cancers and their cultured tumoroid derivatives.

(**A**) Hematoxylin/eosin micrographs of primary tumors not included in Figure 1 (scale=50μm). (**B**) Average number of unique molecular identifiers (UMIs) per cell across scRNA-Seq samples. (**C**) Average number of genes detected per cell across scRNA-Seq samples.

Supplemental Figure 2

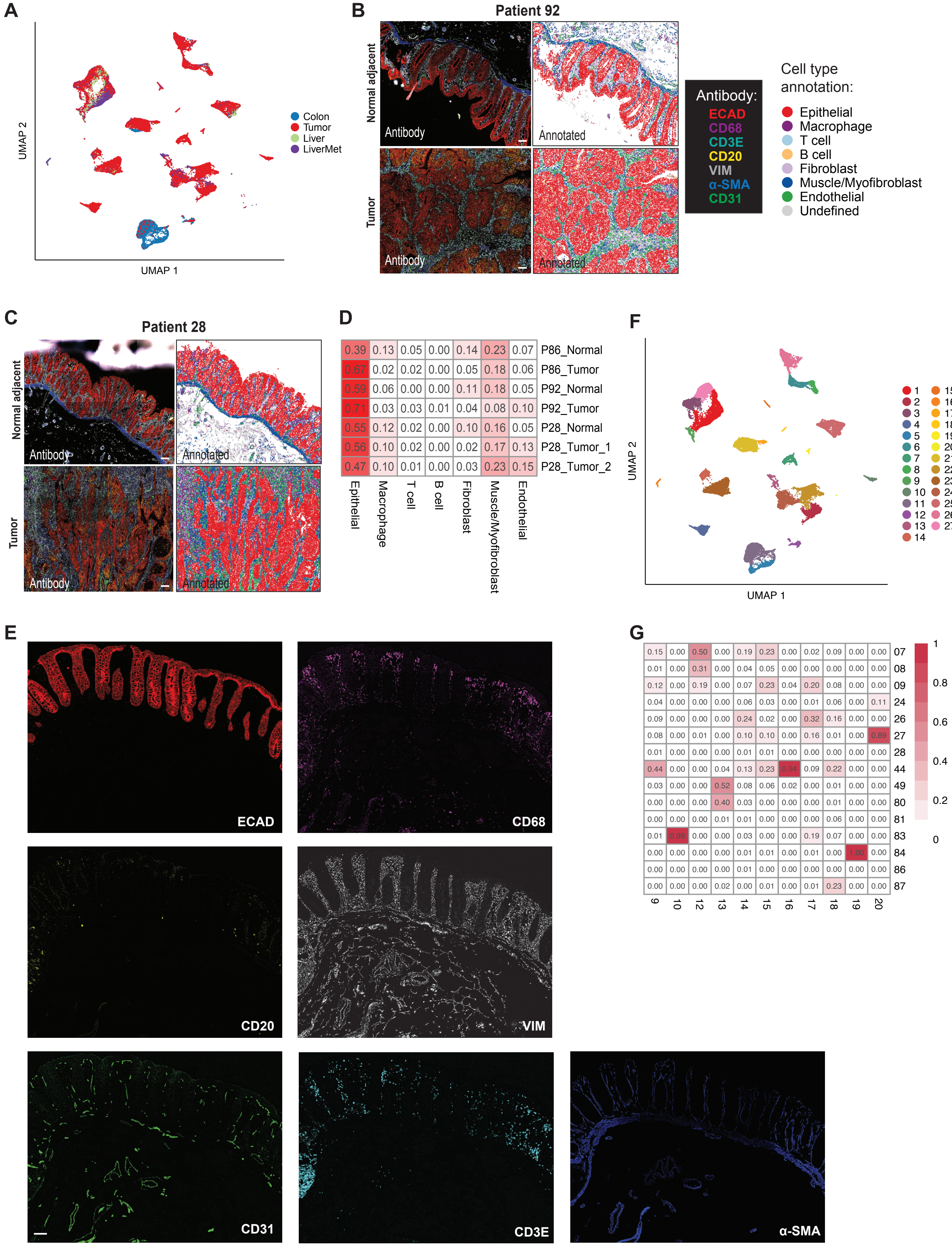

Supplemental Figure 2. Spatial relationships between carcinoma cells and the TME.

(**A**) UMAP as in Fig. 2A, highlighting the source type of the cells. (**B, C**) Left panel: CODEX image of normal adjacent tissues and primary tumor from patient 92 (B) and 28 (C), highlighting seven cell-type markers – ECAD, CD68, CD3E, CD20, VIM, α-SMA and CD31. Right panel: post-segmented image colored by cell type (scale=50μm). (**D**) CODEX-based cell type composition for samples from patient 86 (Fig. 2C), patient 92 (B) and patient 28 (C and for another tumor section not shown here). Value represents proportion of each cell type in the sample normalized by total cell count. (**E**) CODEX images from the normal adjacent colon section from patient 86 shown in Fig. 2, with individual channels separated (scale=50μm). (**F**) Same UMAP colored by Louvain clustering result. (**G**) Sample composition of clusters with less than 1000 cells.

Supplemental Figure 3

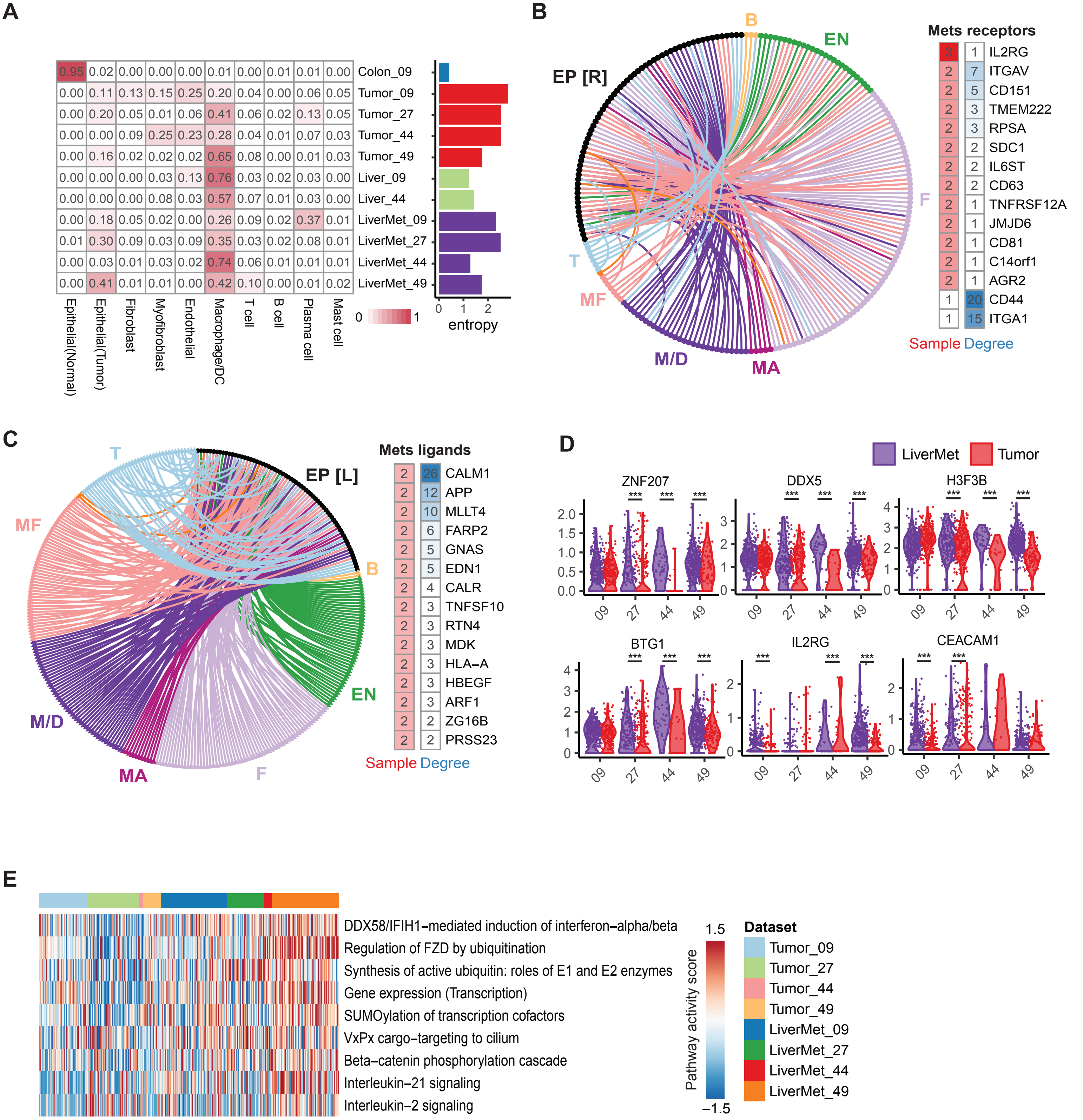

Supplemental Figure 3. Analysis of liver metastasis of human colorectal cancer.

(**A**) Cell type composition of each sample. Only patients with liver metastasis data were included. Value represents proportion of each cell type in the sample (normalized by total cell count instead of epithelial cell count as in Fig. 2B, due to lack of normal colon epithelial cells in the liver samples). For each sample, entropy was calculated based on the cell type proportion to measure the heterogeneity of TME of each sample. (**B**) Receptor-ligand interaction up-regulated in liver metastasis. Each edge indicates communication between a receptor upregulated in metastatic tumor epithelial cells compared to primary tumor epithelial cells, and a ligand expressed by microenvironment cells. Receptors were ranked based on the number of patients in which the gene is differentially expressed, and the degree on the communication graph. EP [R]: receptors expressed on epithelial cells; B: B cells; T: T cells; EN: endothelial cells; F fibroblasts; MF: Myofibroblasts; M/D: Macrophage/dendritic cells. MA: mast cells. (**C**) Same as (B) but highlighting the ligands up-regulated in metastatic tumor epithelial cells compared to primary tumor epithelial cells, and corresponding receptors expressed by microenvironment cells. (**D**) Violin plots of expression level of selected genes that show consistently increased expression in liver metastasis compared to primary tumor across multiple patients. (**E**) Top up-regulated Reactome pathways in liver metastasis. Pathway scores for each cell were computed using AUCell package followed by t-test to derive differentially activated pathways.

Supplemental Figure 4

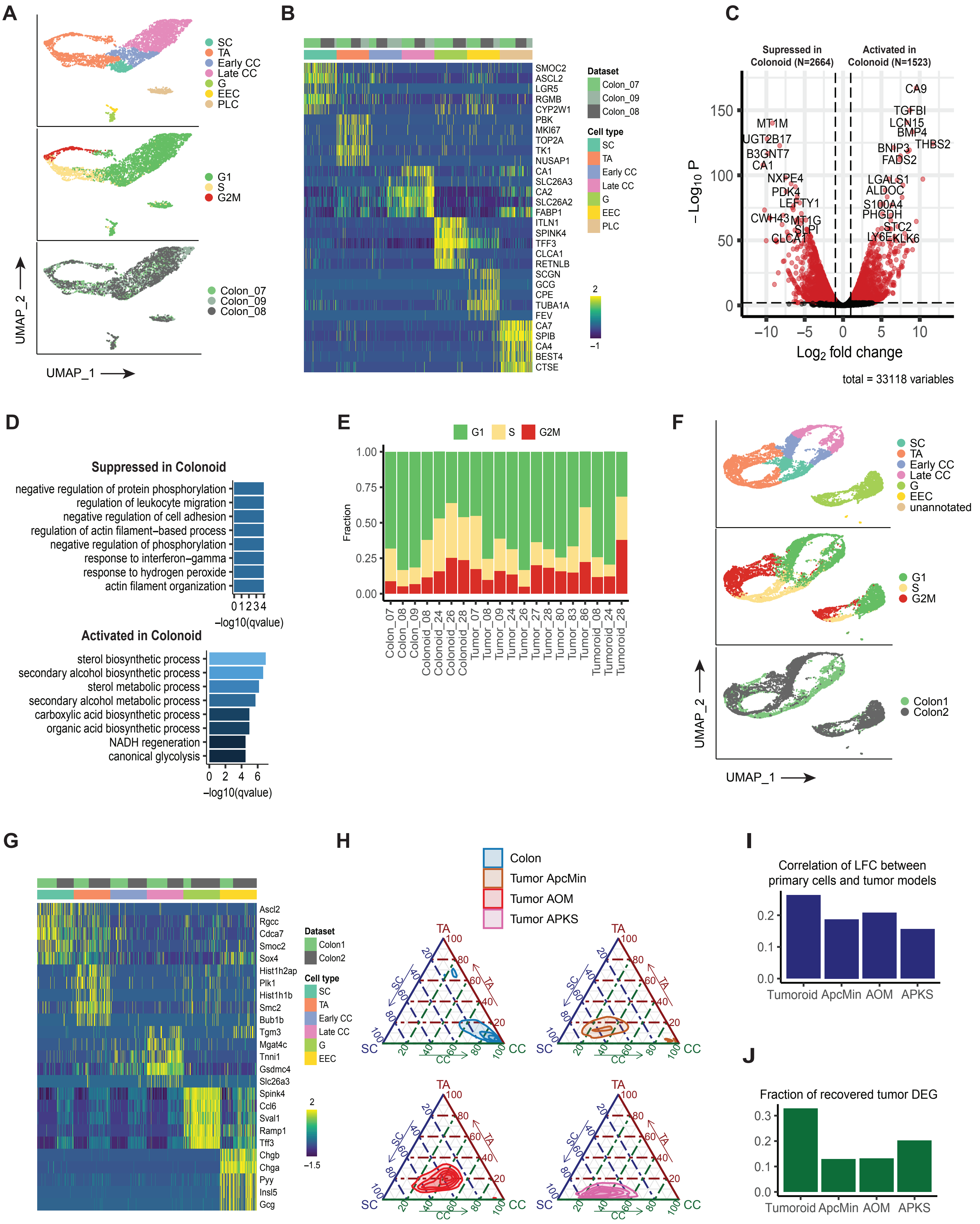

Supplemental Figure 4. Analysis of human carcinoma and normal epithelial cells *in vivo* and *in vitro* with comparison to mouse models of colorectal cancer.

(**A**) UMAP of normal adjacent colon epithelial cells, colored by cell type, cell cycle phase and patient id. SC: stem cells; TA: transit amplifying (TA) cells; Early CC: early colonocytes; Late CC: late colonocytes; G: goblet cells; EEC: enteroendocrine cells; PLC: Paneth-like cells. (**B**) Expression heatmap of top differentially expressed genes of each cell type in human. (**C**) Volcano plot showing genes activated and suppressed in organoid compared to normal adjacent colon tissue based on differential expression analysis. Red point represents genes significantly up- or down-regulated in organoid compared to normal adjacent colon, with FDR ≤ 0.01 and log2 fold change ≥ 1. (**D**) Gene ontology (GO) functional analysis of DEGs activated and suppressed in organoids-log10(q-value) of the top significantly enriched GO terms in biological process were plotted. (**E**) Bar plot showing cell cycle phase composition for each dataset. (**F**) UMAP of mouse normal colon, colored by cell type, cell cycle phase, and replicate id. Cells were merged from two separate scRNA-Seq experiments and display some batch effect. However, both batches showed consistent expression of cell-type marker genes. SC: stem cells; TA: transit amplifying (TA) cells; Early CC: early colonocytes; Late CC: late colonocytes; G: goblet cells; EEC: enteroendocrine cells; PLC: Paneth-like cells. (**G**) Expression heatmap of top differentially expressed genes of each cell type in mouse. (**H**) Ternary plot showing distribution of cells in the cell-type-signature space across normal mouse colon and mouse models of colorectal cancer (ApcMin/+, AOM-DSS, and APKS tumoroid implantation models). Cell type signature genes were derived for each epithelial cell type including stem cell (SC), transit amplifying cell (TA) and colonocytes (CC) using differential expression analysis on normal epithelial cells. Signature scores were calculated using the AUCell package, and linearly rescaled to 0 to 100. 2D kernel density estimation was performed to visualize the cell distribution. (**I**) Correlation between log2 fold change (LFC) of genes differentially expressed in primary human carcinoma cells and LFC of corresponding genes/gene orthologs in human tumoroids or mouse tumor models. (**J**) Bar plot showing fraction of primary-tumor-specific gene/gene orthologs that can be recovered by running DE analysis between carcinoma and normal cells using human tumoroids or mouse tumor models.

Supplemental Figure 5

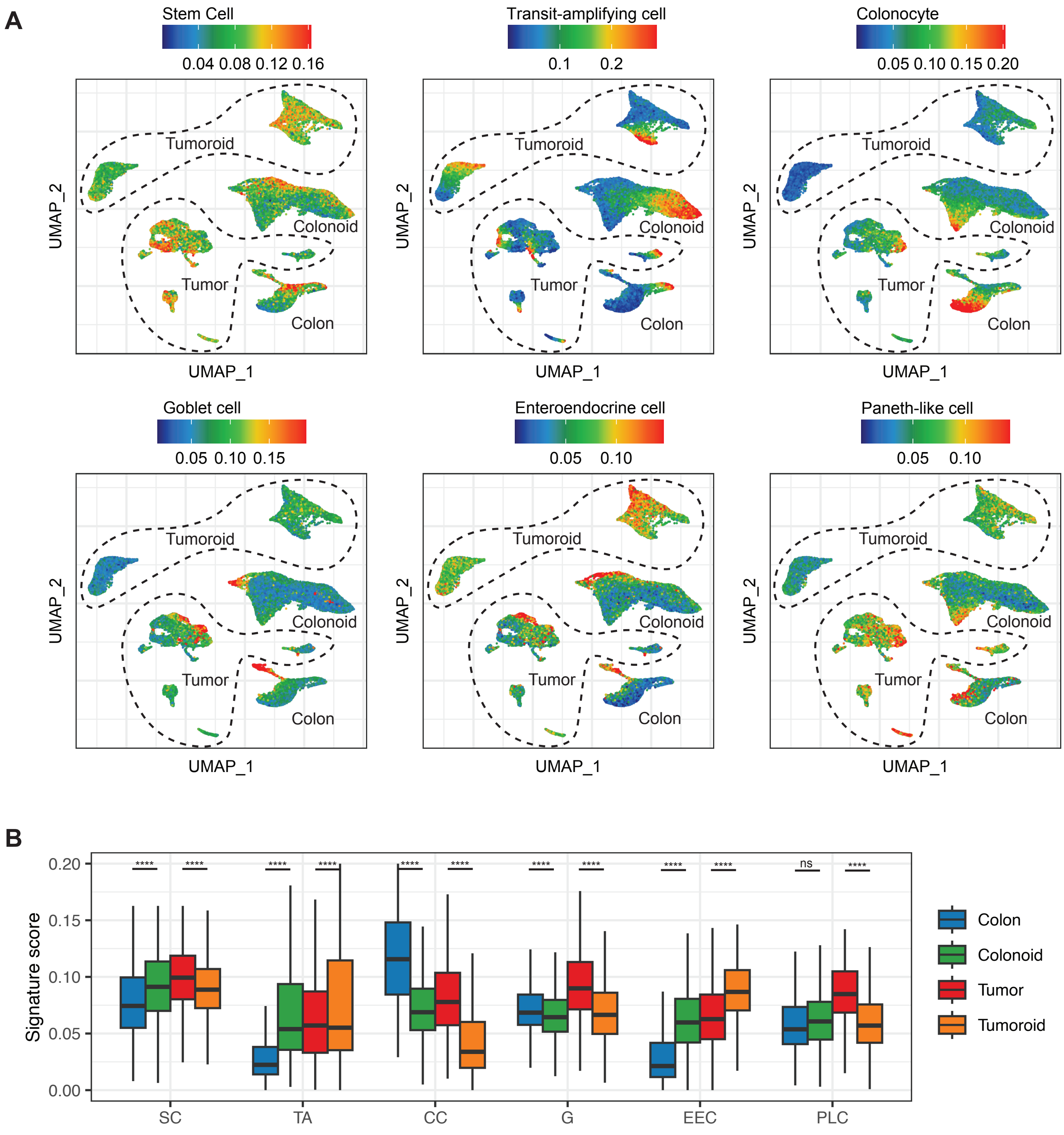

Supplemental Figure 5. Analysis of epithelial cell identity between *in vivo* and *in vitro* samples. (**A**) UMAP of epithelial cells as in Fig. 3A colored by cell type signature scores. Signature scores were calculated using the AUCell package with cell type signature genes, which were derived for each epithelial cell type using differential expression analysis on normal epithelial cells. (**B**) Box plot showing the distribution of epithelial cell type signature score across different sample types. Two-sided student's t test was performed between *in vivo* and *in vitro* conditions. ****$p \leq 0.0001$; ns, $p > 0.05$.

Supplemental Figure 6

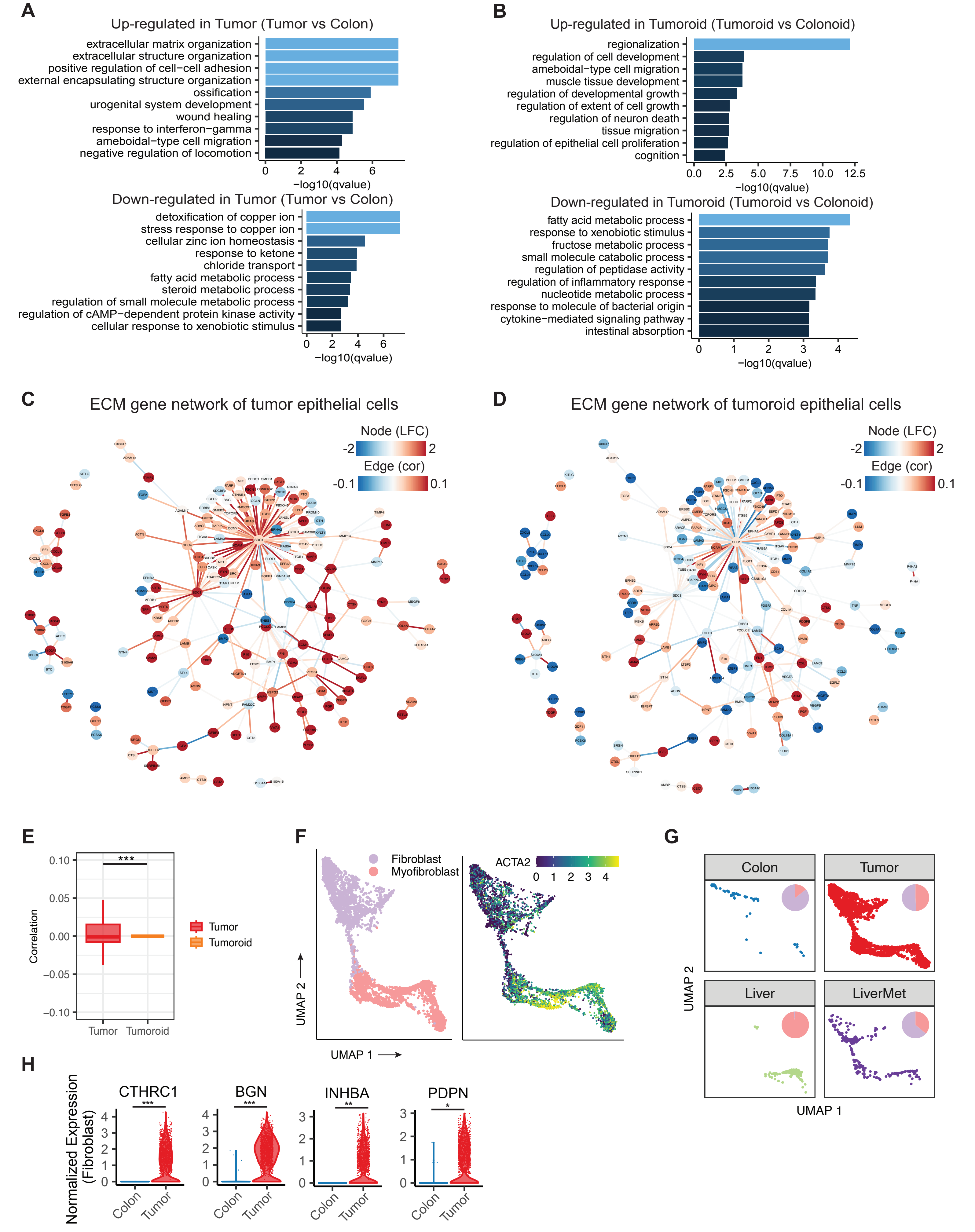

Supplemental Figure 6. Analysis of epithelial-ECM interactions and fibroblast populations. (**A**) GO functional analysis of DEGs differentially expressed in tumor epithelial cells compared to normal epithelial cells. -log10(q-value) of the top significantly enriched GO terms were plotted. (**B**) Same as A but showing top GO terms enriched in genes differentially expressed between tumoroid and normal organoid. (**C**) ECM network from matrixDB, obtained from the matrinetR package[29]. Nodes are colored based on the log2 fold change of DE between tumor and normal epithelial cells. Edges are colored based on pairwise gene expression correlations among tumor epithelial cells. (**D**) Same as C, but with nodes colored based on the log2 fold change of DE between tumoroid and organoid epithelial cells, and edges colored based on pairwise gene expression correlations among tumoroid epithelial cells. (**E**) Correlations between interacting ECM genes in tumor vs tumoroid. One-sided t test was performed with the alternative hypothesis that correlations in the tumor are higher. ***$p \leq 0.001$. (**F**) UMAP isolating fibroblast and myofibroblast populations, with the expression level of *ACTA2* plotted at right. (**G**) Distribution of cells from different sample types on the UMAP, and the relative proportion of fibroblasts and myofibroblasts. (**H**) Expression level of four pro-tumorigenic genes differentially expressed between fibroblasts in normal adjacent tissue vs. CAFs. Significance level is based on adjusted p-value from DE analysis. *$p \leq 0.05$; **$p \leq 0.01$; ***$p \leq 0.001$.

Supplemental Figure 7

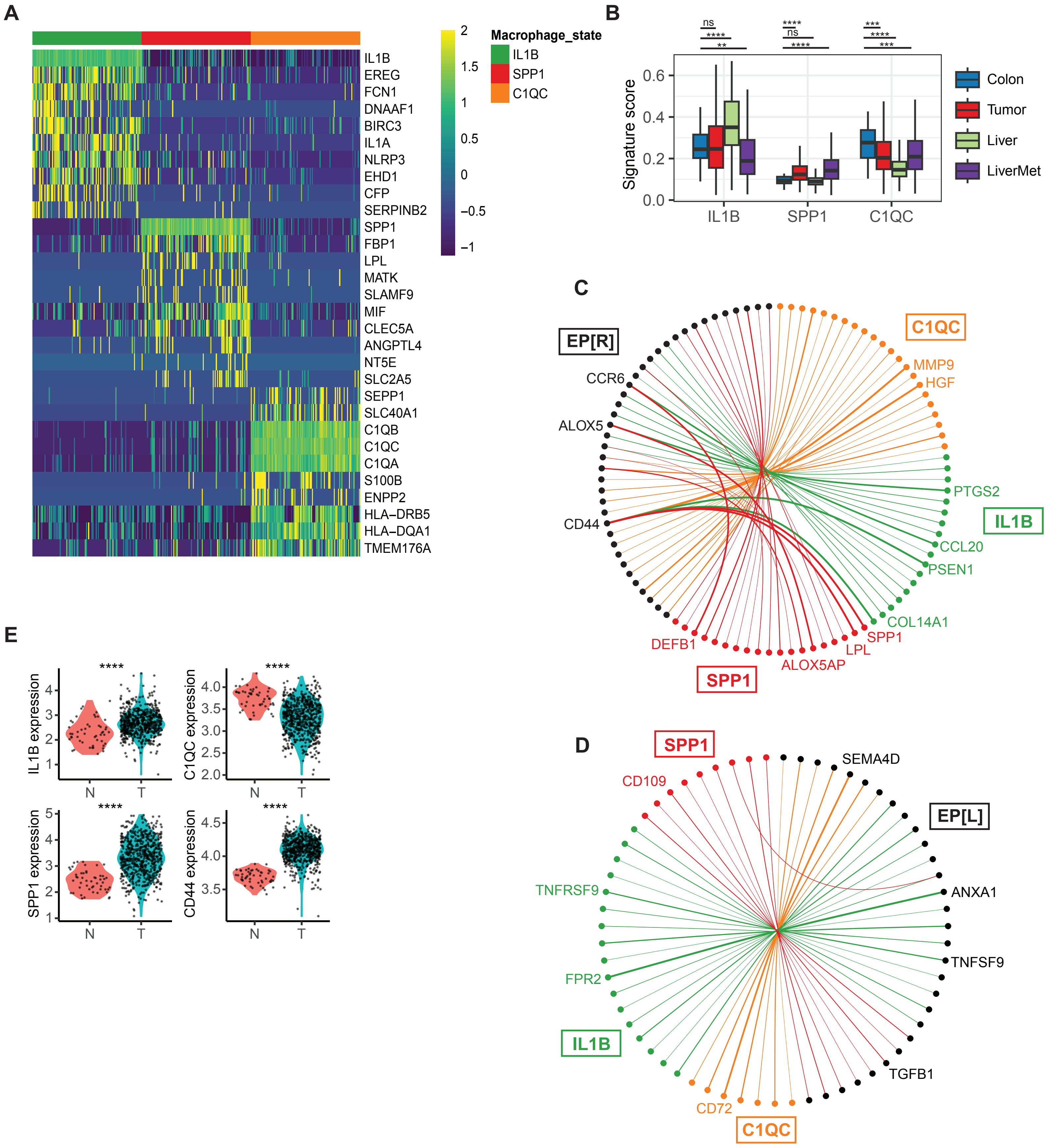

Supplemental Figure 7. Signature genes of macrophage states.

(**A**) Gene expression heatmap of top differentially expressed genes from macrophages in the IL1B, SPP1 and C1QC states. Expression values were log-normalized and z-scored. (**B**) Distribution of signature score of macrophage states across different sample types. Two-sided student's t test was performed between colon and tumor, colon and liver and colon and liver metastasis. **$p \leq 0.01$; ***$p \leq 0.001$; ****$p \leq 0.0001$; ns, $p > 0.05$. (**C**) Receptor-ligand interactions between primary tumor and macrophage subpopulations. Each edge indicates a predicted interaction between a receptor up-regulated in primary tumor carcinoma cells compared to normal adjacent epithelial cells, and a ligand differentially expressed between the macrophage states. Edge widths indicate the number of patients (samples) in which the receptor is significantly up-regulated. EP [R]: receptors expressed on epithelial cells. (**D**) Same as C, but highlighting the ligands up-regulated in primary tumor carcinoma cells compared to normal adjacent epithelial cells, and corresponding receptors differentially expressed by macrophage states. EP [L]: ligands expressed on epithelial cells. (**E**) Expression levels of IL1B, C1QC, SPP1 and CD44 in the TCGA colon (COAD) and rectal (READ) datasets. Two-sided student's t test was performed to compare the expression between tumor and normal samples. ****$p \leq 0.0001$.

Supplemental Figure 8

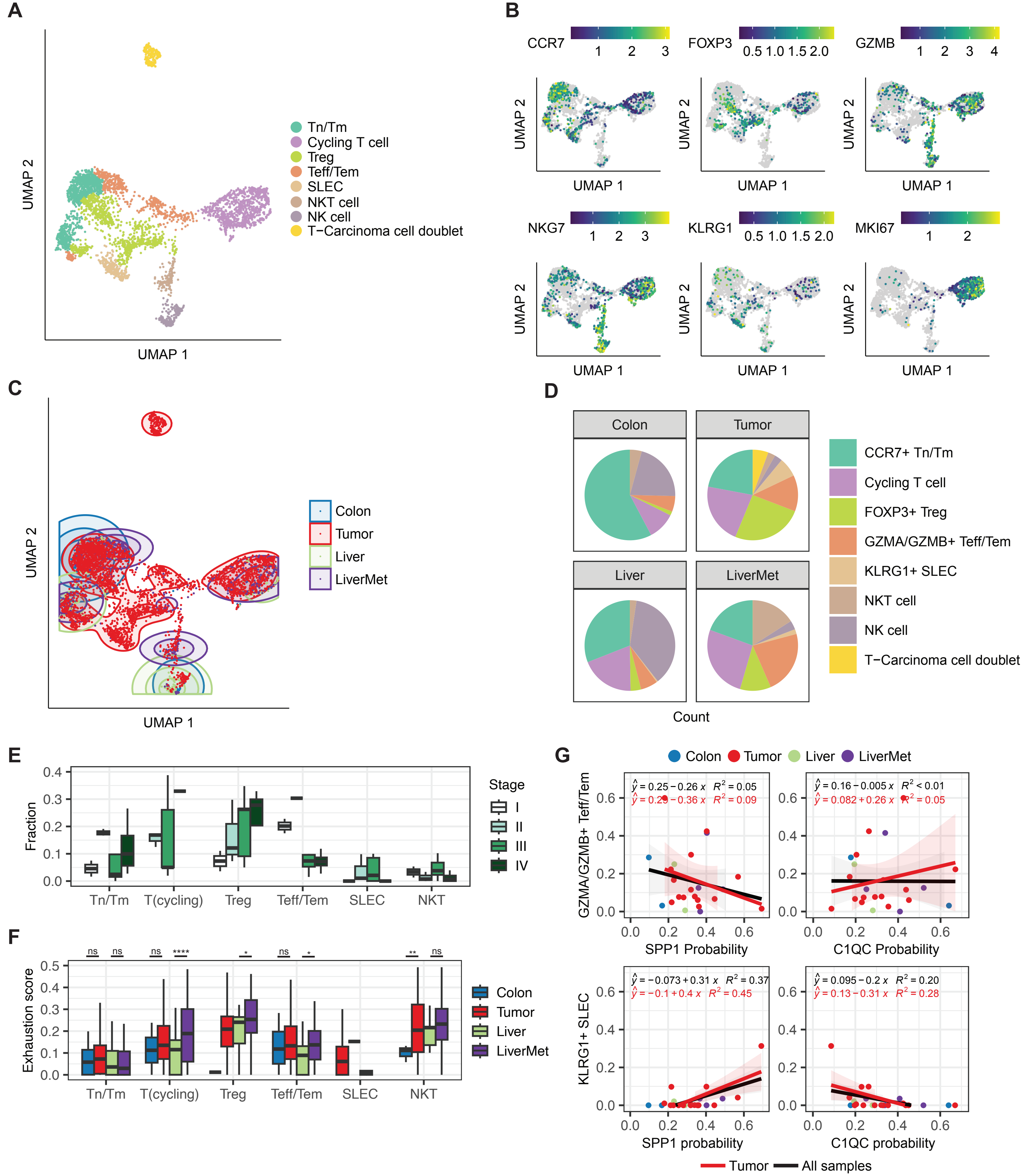

Supplemental Figure 8. Analysis of T and NK cells. (**A**) UMAP of T and NK cells colored by cell subtypes. Tn/Tm: Naive or memory T cell; Treg: regulatory T cell; Teff/Tem: effector or effector memory T cell; SLEC: short-lived effector cell; NKT cell: natural killer T cell; NK cell: natural killer cell. (**B**) UMAP colored by expression level of cell subtype marker genes. (**C**) Distribution of cells in different sample types on the UMAP. (**D**) Cell subtype composition across different sample types. (**E**) Fraction of major T cell subtypes across different tumor stages. MSI samples were excluded. (**F**) Exhaustion score computed based on a curated set of exhaustion marker genes across different T cell subtypes and sample types. Two-sided student's t test was performed between colon and tumor and liver and liver metastasis, omitting conditions with too few cells of that subtype. (**G**) Top two panels show association between Teff/Tem fraction and average macrophage SPP1+ and C1QC+ state probability. Bottom two panels show association between SLEC fraction and average macrophage SPP1+ and C1QC+ state probability. Linear regression was performed for all *in vivo* samples or tumor samples only.

Supplemental Figure 9

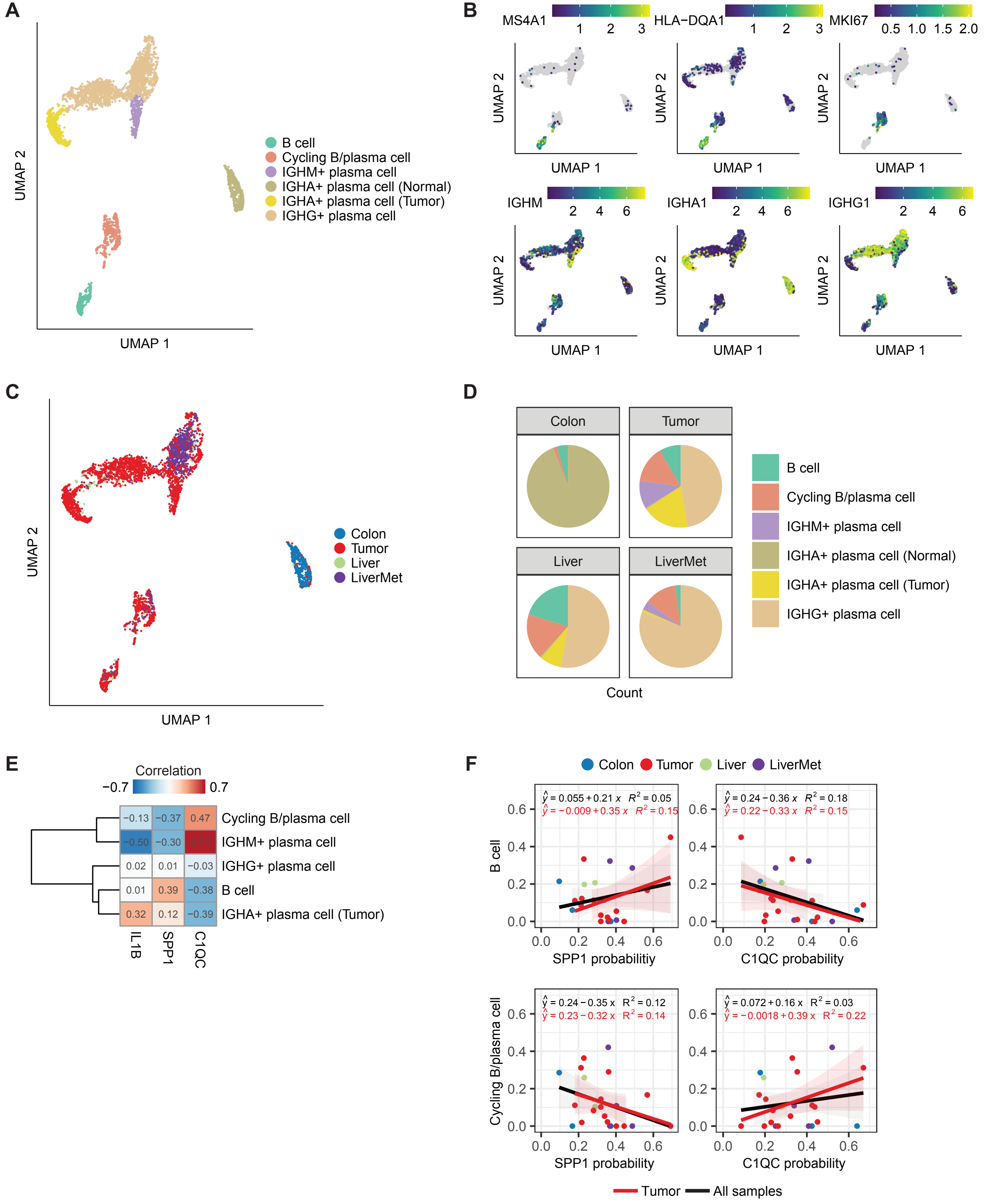

Supplemental Figure 9. Analysis of B cells. (**A**) UMAP of B cells colored by cell subtypes. (**B**) UMAP colored by expression level of B cell subtype marker genes. (**C**) UMAP of B cells colored by sample type. (**D**) B cell subtype composition across different sample types. (**E**) Correlation between the B cell subtype fraction and average macrophage state probability across all *in vivo* samples. (**F**) Top two panels show association between naive B cell fraction and average macrophage SPP1+ and C1QC+ state probability. Bottom two panels show association between cycling B/plasma cell fraction and average macrophage SPP1+ and C1QC+ state probability. Linear regression was performed for all *in vivo* samples or tumor samples only.

Supplemental Figure 10

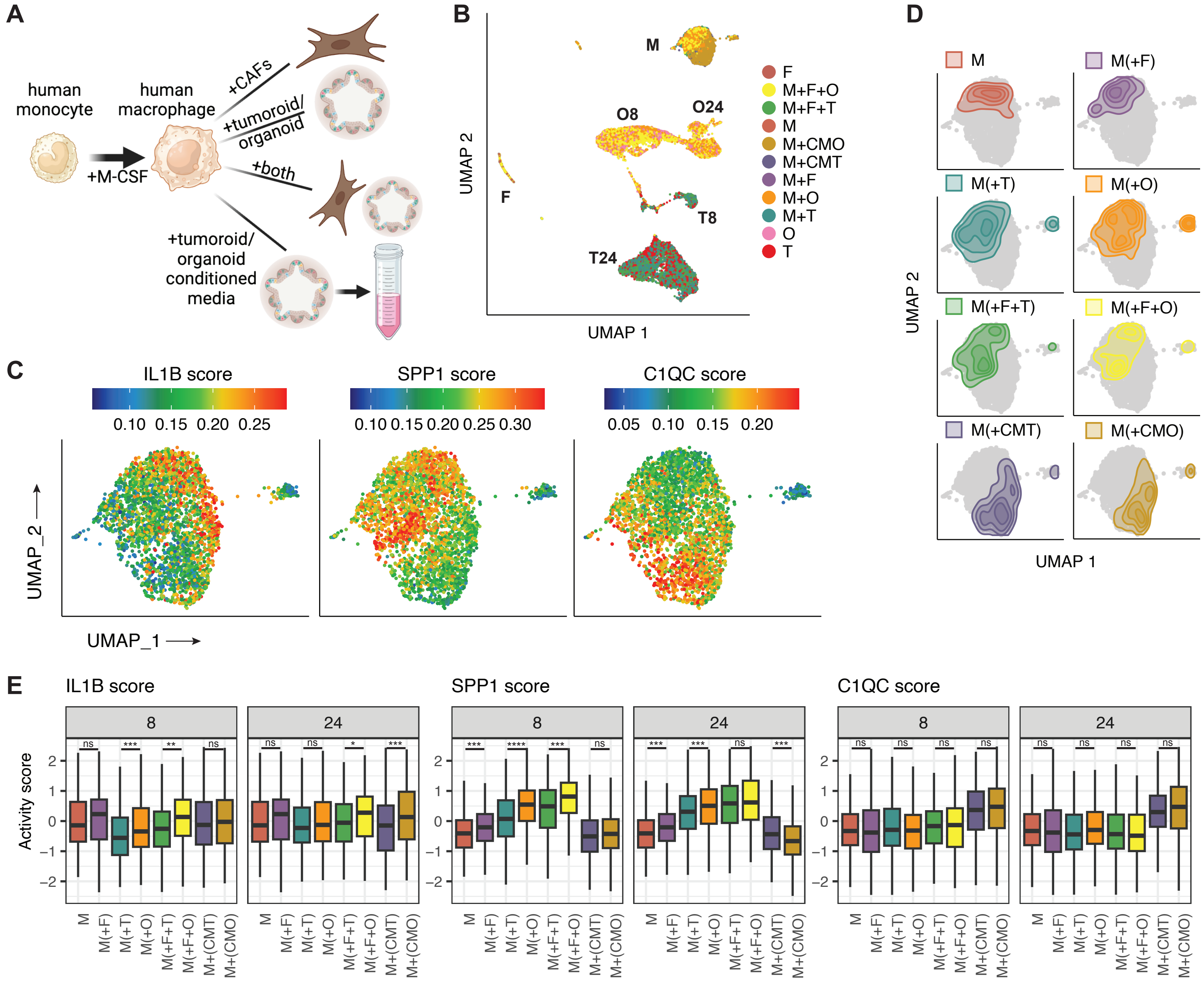

Supplemental Figure 10. Induction of the SPP1+ macrophage state by carcinoma cells and the influence of cancer-associated fibroblasts. (**A**) Illustration of the design of the experiment. (**B**) UMAP of organoid and tumoroid cells (patients 8 and 24), along with macrophages and cancer-associated fibroblasts (CAFs) before and after co-culture. O: normal organoid cells; T: tumoroid cells, M: macrophages; F: cancer associated fibroblasts. CMO: conditional media from normal organoid culture. CMT: conditional media from tumoroid. (**C**) Signature score of macrophage states plotted on the UMAP from (B), viewing only macrophages. The small cluster to the right of the plot are dying macrophages with high mitochondrially-derived transcript levels. (**D**) Distribution of macrophages across different culture conditions on the UMAP. (**E**) Signature scores for macrophage states across conditions and patients. Two-sided student's t test was performed between selected conditions. *$p \leq 0.05$; **$p \leq 0.01$; ***$p \leq 0.001$; ****$p \leq 0.0001$; ns, $p > 0.05$.
