# Supplementary material for: Mapping and modeling human colorectal carcinoma interactions with the tumor microenvironment
Source: Nat Commun. 2023 Nov 30;14:7915. doi: 10.1038/s41467-023-43746-6 (PMC10689473; doi:10.1038/s41467-023-43746-6)
Supplement: Supplementary file 3 — Description of Additional Supplementary Files [file 41467_2023_43746_MOESM3_ESM.pdf]

Description of Additional Supplementary Files

Supplementary Data 1: Potential receptor-ligand interactions between carcinoma cells and cells within their microenvironment.

Supplementary Data 2: Differentially Expressed Genes (DEGs) between Tumor/Tumoroid/Colon/Colonoid cells. DE analysis was carried out using sSeq method. Pvalues were adjusted using the Benjamini & Hochberg method.

Supplementary Data 3: GO enrichment analysis result on DEGs from supplemental table 2, computed using the ClusterProfiler package. P-values were adjusted using the Benjamini & Hochberg method.

Supplementary Data 4: Prediction of Consensus Molecular Subtypes through utilization of the CMScaller R package. P-values were adjusted using the Benjamini & Hochberg method.

Supplementary Data 5: List of CODEX Antibodies and Oligonucleotides.

Supplementary Data 6: Summary of Statistical Tests.
