# Supplementary material for: Mapping and modeling human colorectal carcinoma interactions with the tumor microenvironment
Source: Nat Commun. 2023 Nov 30;14:7915. doi: 10.1038/s41467-023-43746-6 (PMC10689473; doi:10.1038/s41467-023-43746-6)
Supplement: Supplementary file 10 — Reporting Summary [file 41467_2023_43746_MOESM10_ESM.pdf]

nature portfolio

Corresponding author(s): Lengner and Li
NCOMMS-22-42587

Last updated by author(s): Oct 6, 2023

Reporting Summary

Nature Portfolio wishes to improve the reproducibility of the work that we publish. This form provides structure for consistency and transparency in reporting. For further information on Nature Portfolio policies, see our Editorial Policies and the Editorial Policy Checklist.

Statistics

For all statistical analyses, confirm that the following items are present in the figure legend, table legend, main text, or Methods section.

n/a	Confirmed	
☐	☒	The exact sample size (*n*) for each experimental group/condition, given as a discrete number and unit of measurement
☒	☐	A statement on whether measurements were taken from distinct samples or whether the same sample was measured repeatedly
☐	☒	The statistical test(s) used AND whether they are one- or two-sided<br>*Only common tests should be described solely by name; describe more complex techniques in the Methods section.*
☐	☒	A description of all covariates tested
☐	☒	A description of any assumptions or corrections, such as tests of normality and adjustment for multiple comparisons
☐	☒	A full description of the statistical parameters including central tendency (e.g. means) or other basic estimates (e.g. regression coefficient) AND variation (e.g. standard deviation) or associated estimates of uncertainty (e.g. confidence intervals)
☐	☒	For null hypothesis testing, the test statistic (e.g. *F*, *t*, *r*) with confidence intervals, effect sizes, degrees of freedom and *P* value noted<br>*Give P values as exact values whenever suitable.*
☒	☐	For Bayesian analysis, information on the choice of priors and Markov chain Monte Carlo settings
☐	☒	For hierarchical and complex designs, identification of the appropriate level for tests and full reporting of outcomes
☐	☒	Estimates of effect sizes (e.g. Cohen's *d*, Pearson's *r*), indicating how they were calculated

Our web collection on statistics for biologists contains articles on many of the points above.

Software and code

Policy information about availability of computer code

Data collection	Cell Ranger
Data analysis	RStudio, Loupe Browser, Seurat, Monocle3, VisCello

For manuscripts utilizing custom algorithms or software that are central to the research but not yet described in published literature, software must be made available to editors and reviewers. We strongly encourage code deposition in a community repository (e.g. GitHub). See the Nature Portfolio guidelines for submitting code & software for further information.

Data

Policy information about availability of data

All manuscripts must include a data availability statement. This statement should provide the following information, where applicable:

- Accession codes, unique identifiers, or web links for publicly available datasets
- A description of any restrictions on data availability
- For clinical datasets or third party data, please ensure that the statement adheres to our policy

The following secure token has been created to allow review of record GSE203608 while it remains in private status:
obeticgelxetnaj

Research involving human participants, their data, or biological material

Policy information about studies with human participants or human data. See also policy information about sex, gender (identity/presentation), and sexual orientation and race, ethnicity and racism.

Reporting on sex and gender	In total, we collected tumors from 17 patients spanning tumor grade, CMS type, and stage. Both female and male candidates are included in the current study.
Reporting on race, ethnicity, or other socially relevant groupings	No particular attention given to race, ethnicity, or other socially pertinent factors. Patient has not received prior chemotherapy treatment before the surgery and sample collection.
Population characteristics	The ages of the patients span from 42 to 80.
Recruitment	For those individuals who consent, after surgical resection, tumors are received in pathology, grossly examined by a pathologist, and a section of tumor and normal adjacent colon is obtained when extra tissue can be provided for research use without compromising patient care. Tissue is then transferred into ice cold phosphate-buffered saline (PBS) and placed on ice until use.
Ethics oversight	University of Pennsylvania Institutional Review Board

Note that full information on the approval of the study protocol must also be provided in the manuscript.

Field-specific reporting

Please select the one below that is the best fit for your research. If you are not sure, read the appropriate sections before making your selection.

☒ Life sciences ☐ Behavioural & social sciences ☐ Ecological, evolutionary & environmental sciences

For a reference copy of the document with all sections, see nature.com/documents/nr-reporting-summary-flat.pdf

Life sciences study design

All studies must disclose on these points even when the disclosure is negative.

Sample size	We collected tumors from 17 patients in this study.
Data exclusions	All data was included in the studies, except for instances where regrouping was essential for specific analysis.
Replication	Not applicable in the study.
Randomization	No randomization in the study.
Blinding	No blinding was used throughout experiments. All data collected was quantifiable and blinding would not mitigate any subjective biases.

Reporting for specific materials, systems and methods

We require information from authors about some types of materials, experimental systems and methods used in many studies. Here, indicate whether each material, system or method listed is relevant to your study. If you are not sure if a list item applies to your research, read the appropriate section before selecting a response.

Materials & experimental systems

n/a	Involved in the study	
☐	☒	Antibodies
☒	☐	Eukaryotic cell lines
☒	☐	Palaeontology and archaeology
☐	☒	Animals and other organisms
☒	☐	Clinical data
☒	☐	Dual use research of concern
☒	☐	Plants

Methods

n/a	Involved in the study	
☒	☐	ChIP-seq
☒	☐	Flow cytometry
☒	☐	MRI-based neuroimaging

Antibodies

Antibodies used	Akoya antibodies were purchased preconjugated to their respective CODEX barcode. All other antibodies were custom conjugated to their respective CODEX barcode. See full antibody list in Supplemental Table 4.
Validation	Akoya antibodies have been validated by the company. We did purchase preconjugated to their respective CODEX barcode.

Animals and other research organisms

Policy information about studies involving animals; ARRIVE guidelines recommended for reporting animal research, and Sex and Gender in Research

Laboratory animals	C57BL/6J (JAX strain 000664), Apcflox/flox, KrasLSL-G12D (JAX strain 008179)
Wild animals	Not applicable in the study.
Reporting on sex	Sex was not factored into the experimental design, although both genders were incorporated into the project.
Field-collected samples	Not applicable in the study.
Ethics oversight	All procedures involving mice were reviewed and approved by the Institutional Animal Care and Use Committee of the University of Pennsylvania (Animal Welfare Assurance Reference Number no. A3079-01, approved protocol no. 803415 granted to Dr. Lengner) and were in accordance with the guidelines set forth in the Guide for the Care and Use of Laboratory Animals of the National Research Council of the National Institutes of Health.

Note that full information on the approval of the study protocol must also be provided in the manuscript.
